# Clinical Characteristics of Systemic Lupus Erythematosus in Caucasians and Latin American Hispanics: Data from a Single Tertiary Center

**DOI:** 10.1155/2024/5593302

**Published:** 2024-08-27

**Authors:** Luca Marri, Chiara Vassallo, Pasquale Esposito, Luca Bottaro, Raffaele De Palma, Simone Negrini

**Affiliations:** ^1^ Department of Internal Medicine University of Genova, Genova 16132, Italy; ^2^ Internal Medicine Clinical Immunology and Translational Medicine Unit IRCCS Ospedale Policlinico San Martino, Genova 16132, Italy; ^3^ Nephrology Dialysis and Transplantation Unit IRCCS Ospedale Policlinico San Martino, Genova 16132, Italy

## Abstract

**Background:**

Different studies report that systemic lupus erythematosus (SLE) tends to have a more aggressive course in Hispanic patients. In this study, we analysed epidemiologic, clinical, and laboratory characteristics in a cohort of Hispanic and Caucasian lupus patients in the context of Italian health service, which provides free access to care to all citizens, thus mitigating the impact of socioeconomic factors that negatively influence the course of the disease in ethnic minorities.

**Methods:**

This single-center retrospective study was conducted at the San Martino Hospital “Lupus Clinic” in Genoa, Italy. Patients ≥18 years with a confirmed diagnosis of SLE and definite ethnicity (Hispanic or Caucasian) were recruited.

**Results:**

A total of 126 patients (90 Caucasians and 36 Hispanics) were enrolled. We compared epidemiologic characteristics, clinical features, autoantibodies profile, and treatment options without evidencing any statistically significant difference between the two groups, except for disease duration, which was higher in the Caucasian group (20.4 years versus 14.2 years in the Hispanic group, *P*=0.002) and SLICC damage index, which was greater in Caucasian patients (2.11 versus 1.88 in Hispanics, *P*=0.037), but this difference was no longer significant after correction for disease duration (*P*=0.096).

**Conclusions:**

In our cohort, Hispanic ethnicity is not associated with worse disease features and outcomes. Therefore, we speculated that socioeconomic factors, in particular, free access to healthcare, might be more relevant in influencing the course of the disease than genetic background.

## 1. Introduction

Systemic lupus erythematosus (SLE) is a chronic autoimmune disease resulting from a complex interaction of genetic, epigenetic, and environmental favouring factors. The natural history of SLE is typically relapsing-remitting with pleomorphic clinical manifestations potentially involving every organ or tissue [1].

Several studies have shown that some sociodemographic predictors such as ethnicity, gender, age, income, education, and access to healthcare are important variables associated with the epidemiology and the outcome of SLE [2, 3]. In reference to ethnicity, incidence and prevalence rates are consistently higher among those of African, Hispanic, or Asian descent across studies from different countries. Notably, in these same ethnic groups, SLE seems to be more severe with higher disease activity and more damage accrual than in Caucasians [4–6].

Immigration can be considered a relatively new phenomenon in Italy, a country historically characterized by emigration. In the last decades, Genoa, similar to other Italian cities, has experienced a significant increase in immigration, especially from Latin America. According to the Italian National Institute of Statistics (ISTAT), foreign citizens (about 75000) account for 9.1% of the total population of the metropolitan area of Genoa [7]. The most represented foreign community in Genoa are Ecuadorians (around 20% of foreigners), and also other Latin American populations are present in the territory. Globally, Latin American/Hispanic is the most represented non-White ethnicity in our territory [7].

Several studies have pointed out that Hispanics seem to present a higher incidence of SLE, a more severe course of the disease, and poorer outcomes as compared to Caucasians. The LUMINA study was based on a prospective cohort specifically established to analyse minority SLE patients in the United States [8–10]. In this cohort, Texas Hispanics showed greater disease activity, accelerated damage accrual, increased frequency and severity of renal disease, and diminished survival than Caucasians and Puerto Rican Hispanics. Interestingly, Hispanics from Puerto Rico not only have a lesser proportion of ancestral Native American genes but also benefit from better socioeconomic conditions than Texas Hispanics [9, 11–13]. In the GLADEL study, Afro-Latin Americans and Mestizos had more severe SLE and develop renal disease at a higher proportion than Whites [14, 15]. Analysing disease features and outcomes in US SLE patients of Hispanic origin and their Mestizo counterparts in Latin America from the LUMINA and GLADEL cohorts, it emerged that US Hispanics seemed to have a poorer prognosis, despite a comparable genetic background, thus suggesting that socioeconomic factors may account for these discrepancies [16]. Similarly, Mexican patients living in Mexico had lower levels of disease activity and damage with respect to Texan Hispanics from the LUMINA cohort, of whom 95% are of Mexican descent [17]. Calvo-Alén et al. reported that Hispanics with a strong Amerindian background (US Hispano-Americans) have a more serious disease than that observed in Hispanics from Northern Spain, suggesting that the genetic pool and also socioeconomic differences between these two Hispanic subgroups probably explain for these findings [10]. Data from the RELESSER registry confirm this observation indicating that Latin-American Hispanic patients have an increased frequency of lupus nephritis and display higher disease severity than European Caucasians Hispanics [18, 19]. The multiethnic study profile showed that renal involvement is more frequent among non-Caucasian patients, and, within them, Hispanic patients seem to be at a higher risk of renal damage [20, 21]. The CDC, through five different US cohorts, reports that Hispanics have a higher incidence and prevalence of lupus and are at an increased risk of severe manifestations and early development of lupus nephritis, thrombocytopenia, and antiphospholipid syndrome [22].

Ethnicity also impacts drug response. In the short-term induction therapy for lupus nephritis, mycophenolate mofetil (MMF) and intravenous cyclophosphamide (CYC) have a similar overall efficacy; however, a significantly higher response to MMF with respect to CYC was observed in patients of Mestizo descent and individuals of Hispanic origin [23]. In the EXPLORER trial on rituximab in nonrenal lupus, Hispanic patients achieved significantly higher major clinical responses compared with other groups [24]. Unfortunately, limited access to these drugs in several Latin American countries, due primarily to cost issues, or in developed countries, owing to disparities in healthcare access often experienced by racial and ethnic minorities, hinders these patients from accessing the most effective medical care. This aspect is clearly exemplified by the recent GLADEL—Pan-American League of Associations of Rheumatology (PANLAR) recommendations, where not only the efficacy but also the costs and availability of a given medication strongly influence its usage in clinical practice of specific geographical context [25].

Overall, the abovementioned investigations indicate that Hispanics tend to have an increased prevalence, disease severity, risk of complications, and a worse outcome compared to Caucasian patients. Despite the consistent literature on the subject, the explanation of these disparities has not been univocally established, and differences in biologic/genetic background (e.g., higher proportion of ancestral Native American genes) and socioeconomic factors (e.g., unfavourable economic status, education, or access to healthcare) might contribute in a nonmutually exclusive manner.

The purpose of this study was to analyse epidemiological characteristics, clinical and laboratory features, and treatment options in an Italian cohort of SLE patients of Hispanic and Caucasian ethnicity. This study was conducted in the context of the Italian health system which provides universal coverage to all citizens, allowing to mitigate, at least in part, the bias due to socioeconomic disparities in healthcare access that typically impact racial/ethnic minorities.

## 2. Materials and Methods

### 2.1. Study Design

This is a retrospective and cross-sectional single-center study. The study was conducted in compliance with the Declaration of Helsinki and Good Clinical Practice guidelines. The study was approved on October 17, 2022, by the local medical ethical committee (Liguria Regional Ethical Committee registry number 485/2022-DB id 12602), and all patients provided informed consent before the inclusion in the study.

### 2.2. Patients

Patients ≥18 years old with SLE, defined according to the European Alliance of Associations for Rheumatology (EULAR) and the American College of Rheumatology (ACR) 2019 classification criteria (2019 EULAR/ACR) [26], and definite ethnicity (see below) were recruited at San Martino Hospital Lupus Clinic, a dedicated SLE clinic located in Genoa, Italy, between October 2022 and March 2023. Caucasian patients were defined as those born in Italy with a Caucasian phenotype and without known non-Caucasian ancestors. Hispanic patients were defined as those immigrated from Latin American countries with an American-Indian phenotype with Latin American parents and without known African or European ancestors [18]. All Hispanic patients had universal health coverage, as is the case with all Caucasian subjects.

Data were extrapolated from medical records and transferred to a fully deidentified database for statistical analysis. Collected variables included sociodemographic and clinical characteristics, as well as laboratory characteristics.

Clinical manifestations have been defined according to the EULAR/ACR 2019 definition system and organized in various clinical domains (hematologic, neuropsychiatric, mucocutaneous, serosal, musculoskeletal, and renal) [26]. The EULAR/ACR 2019 score has been assessed at disease onset and at the time of enrolment in the study [26]. The 2023 EULAR/ACR Classification Criteria have been adopted in order to define the presence of antiphospholipid syndrome (APS) [27].

Disease activity has been calculated by the Systemic Lupus Erythematosus Disease Activity Index 2000 (SLEDAI-2K) [28]. Low Disease Activity State (LLDAS) was defined as SLEDAI-2K ≤ 4 without activity in major organ systems or new disease activity, Physician Global Assessment (PGA) ≤1, prednisone dose ≤7.5 mg/day, and/or immunosuppressive-biologic-antimalarial drugs on maintenance dose [29]. Accumulation of damage has been ascertained through the Systemic Lupus International Collaborative Clinics/American College of Rheumatology Damage Index (SDI), a score system that records irreversible damage that has occurred since the onset of lupus in 12 different systems [30].

Immunological laboratory tests included antinuclear antibody (assessed on Hep-2 cells by indirect immunofluorescence), double-stranded DNA antibodies (assessed on *Crithidia luciliae* by indirect immunofluorescence), anti-Sm antibodies (by immunoenzymatic assay), antiphospholipid antibodies (i.e., anti-cardiolipin and anti-*β*2-glycoprotein antibodies by immunoenzymatic assay), presence of lupus anticoagulant (LAC), and complement levels.

Current treatment (at last visit) and previous therapeutic regimens were obtained from medical records.

### 2.3. Statistical Analysis

Overall comparisons of the clinical and immunologic categorical variables among the two ethnic groups were performed using cross tabulations, and their significance was assessed by means of the chi-square/Fisher's exact test. Regarding continuous variables, the comparison among the two groups was assessed using parametric (Student's *t*-test) or nonparametric (Mann–Whitney *U* test) test according to the distribution of the analysed variables and the sample size. For some relevant variables, a multivariate analysis was performed using a logistic regression model in order to estimate the adjusted odds ratio of each variable. Spearman's rho coefficient was used to assess correlations. All statistical analyses were performed with Jamovi [31].

## 3. Results

### 3.1. Demographic Factors

Overall, 126 SLE patients were enrolled; 90 of them were Caucasians (71%) and 36 were Hispanics (29%). Among Hispanics, most of them were from Ecuador (64%) and Peru (19%). The remaining percentage of Hispanic patients (17%) were from other geographical areas of Latin America. All Hispanics enrolled in this study were first-generation immigrants. As expected, the majority of patients in both ethnic groups were female, and the mean age at the enrolment was 51.6 and 47.8 years in Caucasians and Hispanics, respectively. Among Hispanic patients, 89% received their SLE diagnosis in Italy, while the remaining percentage were diagnosed in their countries of origin. The comparison of the two ethnic groups showed no significant differences in the univariate analysis for gender and age. The demographic variables are detailed in Table 1.

### 3.2. Clinical and Laboratory Variables

Mean age at disease onset, defined as age of onset of first SLE-related manifestation(s), was similar in Caucasians (31.1 years) and in Hispanic patients (33.6 years). Disease duration was significantly higher in Caucasians (20.4 years) than in the Hispanic group (14.2 years). Among Hispanic patients, 89% (32 out of 36) were diagnosed in Italy. Compared to those diagnosed in their countries of birth (11%), no significant differences in age or disease duration were observed (48.6 ± 9.6 vs. 48.3 ± 20.1 years, *P*=0.894; 13.47 ± 8.5 vs. 20.0 ± 15.9 years, *P*=0.413, respectively).

At onset, overt disease (fulfilling SLE classification criteria) was present in 38.9% and 52.7% of Caucasians and Hispanics, respectively (*P*=0.154), whereas the remaining patients of both groups presented initially as undifferentiated connective tissue disease and later developed additional clinical/laboratory manifestations of complete SLE. The mean ACR/EULAR 2019 score assessed at the time of enrolment (last visit) was similar in the two ethnicities, independently from disease duration.

As shown in Table 2, the cumulative prevalence of clinical manifestations (articular, hematologic, renal, mucocutaneous, neuropsychiatric, and serositis) was comparable among the two ethnic groups. These results were maintained after adjustment for disease duration. The detailed analysis of the hematologic domain showed no significant differences between Caucasians and Hispanics in the prevalence of autoimmune hemolytic anaemia (17.8 vs. 19.4%, *P*=0.804), autoimmune thrombocytopenia (32.2% vs. 19.4%, *P*=0.192), and leukopenia (44.4% vs. 55.6%, *P*=0.259). Similarly, the prevalence of specific lupus skin manifestations showed no significant differences in acute (25.6% vs. 33.3%, *P*=0.379), subacute (6.7% vs. 0%, *P*=0.182), and chronic cutaneous lupus (15.6% vs. 5.6%, *P*=0.151) as well as photosensitivity (24.4% vs. 27.8%, *P*=0.698) in the two ethnicities.

The percentage of patients with renal involvement was not significantly different in Caucasians and Hispanics (62.2% vs. 50.0%, respectively). A kidney biopsy was performed in 54 of the 74 patients who developed renal manifestations (relevant proteinuria and/or increase in the serum creatinine), and histologic LN was confirmed in all samples. The percentage of eligible patients who underwent renal biopsy was similar in the two ethnic groups (71.4% Caucasians vs. 77.8% Hispanics, *P*=0.764). The most common forms of LN observed in our cohort were focal and diffuse proliferative glomerulonephritis (classes III and IV) with no significant differences between the two ethnic groups (77.5% Caucasians vs. 64.3% Hispanics, *P*=0.479).

As concern immunological markers of disease, no significant differences in the prevalence of anti-dsDNA and anti-Sm antibodies were found across the two ethnic groups (Table 2). Complement reduction (C3 and/or C4 decreased below the lower laboratory limit) was found in the majority of patients in both populations (Table 2) and associated with a higher prevalence of hematologic manifestations (OR, 5.51, 95% CI, 2.16–14.1, *P* < 0.001). Caucasians and Hispanics tested positive for antiphospholipid antibodies in at least two determinations in about one-third of patients of each group. Of those, 42.9% Caucasians and 46.2% Hispanics developed clinical manifestations (thrombotic and/or obstetric events), thus fulfilling the criteria for APS with no difference among the two ethnicities (Table 2).

As concern disease activity, LLDAS was assessed at the last clinical evaluation, with no significant differences between Caucasians and Hispanics (respectively, 88.9% vs. 80.6%, *P*=0.252). The SDI scores, calculated at the time of enrolment, were significantly higher in Caucasian than in Hispanic patients (2.11 ± 1.91 and 1.23 ± 1.31, respectively; *P*=0.037) in the univariate analysis. As expected, we found a correlation between disease duration and SDI score (rho = 0.227, *P*=0.023). Accordingly, the difference in the SDI score between Caucasians and Hispanics lost statistical significance after adjustment for disease duration (*P*=0.096 in the multivariate analysis).

### 3.3. Therapy

During the course of the disease, all patients received different treatment associations including corticosteroids (CCs) and/or hydroxychloroquine (HCQ) and/or disease-modifying antirheumatic agents (conventional synthetic and/or biologic DMARDs). A comparison of the two ethnic groups showed no significant differences in treatment strategies employed (Table 3). Regarding treatments evaluated during the last visit, most of the patients were taking low dose of CCs, defined as an equivalent dose of prednisone ≤7.5 mg/day, with no difference in the two ethnicities. Similarly, the percentage of Caucasian and Hispanic patients on antimalarials and/or DMARDs was comparable in the two groups (Table 3).

## 4. Discussion

In this study, we analysed demographics, clinical, and laboratory features of a cohort of patients affected by SLE, comparing data from Caucasians and Hispanics.

Regarding demographic factors, the two groups were homogeneous in terms of gender and age. As expected, the majority of Hispanic patients were from Ecuador, the most represented foreign community in Genoa [7]. In the current literature, Hispanic patients originate from many different countries (e.g., multicentric studies) or from a single specific country (nation-based single-center studies) or country of origin is simply not reported. Since it is now clear that not all “Hispanics” are the same, our data, derived mainly from Ecuadorian patients, might not be directly compared with other studies [32]. Importantly, Ecuador's population shows high degrees of Native American genetic ancestry [33], a genetic background evoked as an independent factor in SLE onset and severity [2, 34–36]; thus, it might be considered highly representative of the Hispanic patient population.

With regard to clinical characteristics, disease duration was significantly longer in Caucasian than in Hispanic patients (21 vs. 14 years, respectively). This observation may be explained by the fact that immigration is a recent phenomenon in our region and, consequently, our cohort of Hispanic patients includes subjects with a more recent diagnosis of SLE compared to the Caucasian cohort.

The cumulative incidence of clinical manifestations related to SLE, including articular, hematologic, renal, and neuropsychiatric disorders, and serositis as well as mucocutaneous lupus, was evaluated in Hispanic and Caucasian patients without evidencing significant differences in the two ethnicities, even after adjustment for disease duration.

Different studies report a higher prevalence and severity of renal disease in non-Caucasian patients, including Hispanics [9, 11, 12, 14, 15, 18–22]. In our cohort, we did not find significant differences in terms of prevalence of LN, percentage of biopsies performed, and histological classes among the two groups.

Serositis and cytopenias (in particular, thrombocytopenia and leukopenia) are reported in the literature as more frequent in Hispanics than in Caucasians, although to a lesser extent than renal damage [6, 12, 20]. In our study, both populations exhibit a similar prevalence of serosal and hematologic complications, with the latter encompassing single manifestations such as leukopenia, thrombocytopenia, or autoimmune hemolytic anaemia.

In relation to immunologic laboratory features, such as anti-dsDNA antibodies, anti-Sm antibodies, and complement reduction, no differences emerged comparing the two ethnicities. In agreement with what was reported in the literature, complement reduction was correlated with a higher prevalence of hematologic manifestations [37, 38].

Antiphospholipid syndrome (APS) is characterized by vascular thrombosis, gestational morbidity, and/or nonthrombotic manifestations in the presence of antiphospholipid antibodies [39]. According to the literature, antiphospholipid antibodies can be detected in up to 40% of SLE patients, but only one-third of them will develop clinical manifestations with no clear differences among various races/ethnicities [40]. Consistently, in our cohort, we find a comparable prevalence of aPL positivity and APS, with no differences between Caucasian and Hispanic SLE patients.

Several authors have reported that Hispanics with SLE experience higher disease activity and disease-related damage than Caucasians [9, 11, 12, 18, 19, 41]. Regarding disease activity, we assessed LLDAS at the last clinical evaluation, finding no significant differences between the two ethnic groups. Overall, we found that a large proportion of patients in both groups achieved LLDAS at the time of this study. This observation may be explained, at least partially, by the long disease duration characterizing our cohort (globally 19.5 years) compared to patients' populations analysed in other studies. Indeed, it is well known that SLE tends to be more active in the first years after disease onset, while patients with long disease duration show a higher prevalence of low disease activity and remission [42–44]. Damage accrual related to SLE, assessed by the SDI score, was higher in Caucasians, but this difference disappeared after adjustment for disease duration. This observation is not surprising since the damage is correlated to disease duration which, in our cohort, was significantly higher in Caucasians [45].

Both Hispanics and Caucasians received comparable therapeutic options during the course of the disease. As widely recommended, the majority of patients were treated with hydroxychloroquine, a drug characterized by multiple positive effects in SLE with an optimal safety profile [46]. In our cohort, a substantial proportion of patients either discontinued or were on low-dose steroid therapy, to achieve and maintain this result, and the use of steroid-sparing agents, such as immunomodulating-immunosuppressive drugs, is essential [46]. In many countries, the primary constraint in prescribing certain immunosuppressants is related to their cost. In our cohort, due to the policy of the Italian healthcare system, the use of immunosuppressive drugs relies primarily on medical judgement, independent of patients' economic conditions, thus granting the optimal treatment strategy for all patients.

As mentioned, several studies have reported that SLE tends to present a more severe course in Hispanics compared to Caucasians. Unfortunately, the frequent association between non-Caucasian ethnicities and unfavourable socioeconomic status makes biologic and environmental contributions difficult to disentangle. The existing data in the literature predominantly stem from studies conducted in countries where migration is a long-standing phenomenon. Consequently, these findings may not readily translate to countries with a more recent history of immigration, such as Italy. In addition, the majority of these studies are from the American continent where the socioeconomic status of minorities, including access to healthcare, may significantly differ compared to European countries, especially Italy, where the healthcare system provides universal coverage and a largely free access to care to all citizens. To the best of our knowledge, our study is the first investigation conducted in Italy on this subject, and the second one in Europe alongside the Spanish registry RELESSER [18, 19].

There are limitations in this study, which necessarily inform the interpretation of our results. Firstly, the power of our study is limited by the retrospective design, monocentric nature, and the relatively small sample size. The low number of patients might have led to a type II statistical error, and data derived from a single center may limit their generalizability and external validity. Furthermore, the cross-sectional and retrospective design inherently limits our ability to establish causal relationships and introduces potential bias due to the heterogeneity of the studied populations, particularly regarding variations in disease duration. This variability may indeed influence the expression of time-related clinical manifestations and consequently affect the observed results. Nevertheless, the monocentric structure of our study might potentially provide more homogeneous and uniform data as compared to multicentric cohorts. As noted above, all Hispanic SLE patients enrolled in this study were first-generation immigrants. This may represent a limitation of this study related to a bias known as “healthy migrant effect,” a theory postulating that healthy people are more likely to migrate and consequently first-generation immigrants are healthier than the average person in both the home and host countries [47, 48]. The fact that the majority of Hispanic patients have been diagnosed in Italy may be attributed to this phenomenon. The healthy migrant effect could further account for the observation that our cohort of Hispanic patients have a milder-than-expected disease course, similar to that observed in Caucasians, implying that patients with early-onset SLE and/or more severe disease might have been unable to migrate. Interestingly, data from GLADEL have shown that older age at diagnosis is associated with a less severe course of disease [3, 49]. An additional limitation of our study is the omission of potentially relevant variables such as comorbidities or socioeconomical aspects (i.e., yearly income, years of education, home ownership, and home density). However, according to the literature, these elements tend to be unfavourable in Hispanic immigrants and are evoked as an explanation for ethnicity-related differences among SLE patients, differences that were not observed in our study [32, 50].

Hispanic ethnicity is considered an independent risk factor for severe disease and adverse outcomes in SLE, and many authors have emphasized the important, sometimes prominent, role of unfavourable socioeconomic factors to explain these observations [13, 16, 51–53]. In many studies, particularly those conducted in developed countries, Hispanic patients represent an ethnic minority that often suffers from poor socioeconomic conditions, which have a negative impact on access to healthcare. In contrast to these reports, our cohort of patients benefit from free access to a specialized care center that provides comprehensive care and all available treatment options for SLE, independently from their socioeconomic situations.

In conclusion, we observed similar disease features in Hispanics and Caucasians, suggesting that socioeconomic variables, specifically healthcare access, might be more influential on disease course than biologic and genetic background linked to ethnicity. Validation and replication of our findings in larger studies conducted in similar public health settings are crucial for improving our understanding of the role of ethnicity in SLE.

## Figures and Tables

**Table 1:** Sample demographics.

	Global	Caucasian	Hispanic	*P* value
Population	126	90 (71.4%)	36 (28.6%)	
Country of birth				
Italy	90	90 (100%)	—	
Ecuador	23	—	23 (63.9%)	
Peru	7	—	7 (19.4%)	
Chile	1	—	1 (2.8%)	
Argentina	1	—	1 (2.8%)	
Colombia	1	—	1 (2.8%)	
Cuba	1	—	1 (2.8%)	
Dominican Republic	1	—	1 (2.8%)	
Paraguay	1	—	1 (2.8%)	
Gender				
Male	9 (7.1%)	8 (8.9%)	1 (2.8%)	0.444
Female	117 (92.9%)	82 (91.1%)	35 (97.2%)	0.444
Age (years)	50.6 (±12.2)	51.6 (±12.6)	47.8 (±10.8)	0.113

Categorical parameters are given as *n* (%). Continuous variables are given as mean ± SD.

**Table 2:** Clinical and laboratory characteristics.

	Global (*n* = 126)	Caucasian (*n* = 90)	Hispanic (*n* = 36)	*P* value
Age of onset (years)	31.8 (±13.2)	31.1 (±13.6)	33.6 (±12.1)	0.156
Disease duration (years)	18.6 (±10.5)	20.4 (±10.4)	14.2 (±9.44)	**0.002**
Clinical manifestations				
Articular	88 (69.8%)	63 (70.0%)	25 (69.4%)	0.951
Hematologic	79 (62.7%)	56 (62.2%)	23 (63.9%)	0.861
Neuropsychiatric	11 (8.7%)	7 (7.8%)	4 (11.1%)	0.509
Mucocutaneous	55 (43.7%)	38 (42.2%)	17 (47.2%)	0.609
Renal	74 (58.7%)	56 (62.2%)	18 (50.0%)	0.208
Antiphospholipid syndrome	18 (14.3%)	12 (13.3%)	6 (16.7%)	0.779
Serositis	33 (26.2%)	20 (22.2%)	13 (36.1%)	0.109
Laboratory data				
Anti-dsDNA	78 (61.9%)	60 (66.7%)	18 (50%)	0.082
Anti-Sm	20 (15.9%)	12 (13.3%)	8 (22.2%)	0.280
Antiphospholipid positivity	41 (32.5%)	28 (31.1%)	13 (36.1%)	0.588
Low C3 and/or C4	103 (81.7%)	75 (83.3%)	28 (77.8%)	0.456
Disease burden				
ACR/EULAR Score 2019 at last visit	23.1 (±8.02)	23.8 (±7.81)	21.4 (±8.41)	0.128
LLDAS at last visit	109 (86.5%)	80 (88.9%)	29 (80.6%)	0.252
SLICC Damage Index (SDI) at last visit	1.88 (±1.81)	2.11 (±1.91)	1.23 (±1.31)	**0.037**

Categorical parameters are given as *n* (%). Continuous variables are given as mean ± SD. *P* values < 0.05 in bold. ACR/EULAR, American College of Rheumatology-European Alliance of Associations for Rheumatology; dsDNA, double-stranded DNA; LLDAS, lupus low disease activity state; SLICC, Systemic Lupus Erythematosus International Collaborating Clinics. Antiphospholipid positivity was defined as the presence of any of the following: anti-cardiolipin (IgM and/or IgG) and/or anti-*β*2-glycoprotein (IgM and/or IgG) antibodies and/or presence of lupus anticoagulant (LAC).

**Table 3:** Previous and current treatments.

	Global (*n* = 126)	Caucasian (*n* = 90)	Hispanic (*n* = 36)	*P* value
Previous therapies				
Corticosteroids	121 (96.0%)	86 (95.6%)	35 (97.2%)	1.000
Hydroxychloroquine	106 (84.1%)	73 (81.1%)	33 (91.7%)	0.183
DMARDs	103 (81.7%)	73 (81.1%)	30 (83.3%)	1.000
Belimumab	18 (14.3%)	12 (13.3%)	6 (16.7%)	0.779
Current therapy				
Corticosteroids				
PDN ≤7.5 mg/day	114 (90.5%)	83 (92.2%)	31 (86.1%)	0.321
PDN >7.5 mg/day	12 (9.5%)	7 (7.8%)	5 (13.9%)	0.321
Hydroxychloroquine	93 (73.8%)	63 (70.0%)	30 (83.3%)	0.178
DMARDs	63 (50.0%)	43 (47.8%)	20 (55.6%)	0.430
Belimumab	17 (13.5%)	12 (13.3%)	5 (13.9%)	1.000

DMARDs, disease-modifying antirheumatic drug; PDN, prednisone.

## Data Availability

The datasets generated and/or analysed during the current study are not publicly available due to individual data privacy but may be available from the corresponding author on reasonable request, in compliance with ethical and legal standards.
